# Aberrant evoked calcium signaling and nAChR cluster morphology in a *SOD1* D90A hiPSC-derived neuromuscular model

**DOI:** 10.3389/fcell.2024.1429759

**Published:** 2024-06-20

**Authors:** Nathalie Couturier, Sarah Janice Hörner, Elina Nürnberg, Claudio Joazeiro, Mathias Hafner, Rüdiger Rudolf

**Affiliations:** ^1^ CeMOS, Mannheim University of Applied Sciences, Mannheim, Germany; ^2^ Interdisciplinary Center for Neurosciences, Heidelberg University, Heidelberg, Germany; ^3^ Center for Molecular Biology, Heidelberg University, Heidelberg, Germany; ^4^ Institute of Molecular and Cell Biology, Mannheim University of Applied Sciences, Mannheim, Germany; ^5^ Institute of Medical Technology, Mannheim University of Applied Sciences and Heidelberg University, Mannheim, Germany

**Keywords:** acetylcholine receptors, amyotrophic lateral sclerosis, hiPSC, motor neurons, myogenesis, neuromuscular junction, skeletal muscle cells, stem cells

## Abstract

Familial amyotrophic lateral sclerosis (ALS) is a progressive neuromuscular disorder that is due to mutations in one of several target genes, including *SOD1*. So far, clinical records, rodent studies, and *in vitro* models have yielded arguments for either a primary motor neuron disease, or a pleiotropic pathogenesis of ALS. While mouse models lack the human origin, *in vitro* models using human induced pluripotent stem cells (hiPSC) have been recently developed for addressing ALS pathogenesis. In spite of improvements regarding the generation of muscle cells from hiPSC, the degree of maturation of muscle cells resulting from these protocols has remained limited. To fill these shortcomings, we here present a new protocol for an enhanced myotube differentiation from hiPSC with the option of further maturation upon coculture with hiPSC-derived motor neurons. The described model is the first to yield a combination of key myogenic maturation features that are consistent sarcomeric organization in association with complex nAChR clusters in myotubes derived from control hiPSC. In this model, myotubes derived from hiPSC carrying the *SOD1* D90A mutation had reduced expression of myogenic markers, lack of sarcomeres, morphologically different nAChR clusters, and an altered nAChR-dependent Ca^2+^ response compared to control myotubes. Notably, trophic support provided by control hiPSC-derived motor neurons reduced nAChR cluster differences between control and *SOD1* D90A myotubes. In summary, a novel hiPSC-derived neuromuscular model yields evidence for both muscle-intrinsic and nerve-dependent aspects of neuromuscular dysfunction in *SOD1*-based ALS.

## 1 Introduction

Amyotrophic lateral sclerosis (ALS) is an adult-onset neuromuscular disorder which is characterized by progressive muscle atrophy and weakness and a survival time after diagnosis of two to 4 years (Longinetti and Fang, 2019). While most cases of ALS are sporadic, about 10% of patients develop a familial and genetically inherited form of the disease. The first causative gene of ALS, *SOD1*, was identified in 1993 (Rosen et al., 1993). This ubiquitously expressed protein is a copper/zinc superoxide dismutase and catalyzes the inactivation of toxic superoxide into oxygen and hydrogen peroxide. Mutations in the *SOD1* gene might result in an aberrant redox chemistry through either loss or gain of protein function, and give rise to a wide range of cellular alterations (Kaur et al., 2016). Regarding the pathogenesis of ALS, diverse hypotheses have been proposed. For many years, ALS was considered a classical motor neuron disease. According to the “dying-forward” hypothesis, ALS would initiate at the level of upper motor neurons, followed by progressive involvement of lower motor neurons and muscle fibers, ultimately leading to muscle weakness due to lack of innervation and trophic support (Eisen et al., 2024). However, although the loss of motor neurons is an undisputed component of ALS, several experimental and clinical findings advocate for a pleiotropic or a more muscle-centered scenario. Concerning a pleiotropic or non-neuronal cell autonomous origin, experimental models delivered evidence for a causal role of astrocytes (van Damme et al., 2007; Birger et al., 2019; Varcianna et al., 2019; Rajpurohit et al., 2020; Afonso et al., 2023), microglia (Boillée et al., 2006; Christoforidou et al., 2020; Vahsen et al., 2023), and skeletal muscle fibers in motor neuron decay. Notably, some of these models used conditional transgenes expressing mutated proteins only in the cell type of interest and still led to motor neuron decay and ALS-like phenotypes. Regarding the more muscle-centered scenario, defects in skeletal muscles were observed prior to motor neuron death in *SOD1* ^G93A^ mice (Loeffler et al., 2016). Moreover, various cellular and molecular mechanisms were found to be altered in ALS skeletal muscle and satellite cells, independent of any neuronal component, such as mitochondrial and RNA metabolism, proteostasis, and neuromuscular-related signaling (Dobrowolny et al., 2018b; Quessada et al., 2021; Anakor et al., 2022a; Shefner et al., 2023). Recently, also the skeletal muscle secretome has been incriminated as its contents were shown to induce neurotoxicity and/or loss of neurotrophic factors in the ALS condition (Anakor et al., 2022b; Le Gall et al., 2022; Afonso et al., 2023; Stella et al., 2023), underpinning a multisystem pathogenesis relying on dysfunctional intercellular communication. Specifically, and besides the concept of skeletal muscle cells as a source of vesicle-mediated toxicity, the neuromuscular synapse might be of prime importance in ALS progression as these specialized structures are located at the interface of motor neurons and skeletal myofibers. As reported previously, altered skeletal muscle mechanisms supporting neuromuscular junction (NMJ) integrity might contribute to motor neurons demise (Dobrowolny et al., 2018b; Shefner et al., 2023), supporting a critical involvement of muscle cells in the genesis of ALS.

Mutant-*SOD1* mouse models have been used extensively to investigate ALS pathology, but drawbacks associated with animal models led to difficulties in translation to human pathology. To bridge the gap, *in vitro* models have been developed as recently reviewed (Zhou et al., 2023). Human induced pluripotent stem cells (hiPSC) can be exploited for a deeper understanding of developmental biology, disease modeling, and testing of drugs or therapeutic efficiency (Okano et al., 2023) and are therefore valuable tools for addressing ALS pathogenesis. Pioneered by Takahashi and Yamanaka (Takahashi and Yamanaka, 2006), iPSC technology has experienced rapid development, eliciting hiPSC determination towards various lineages. However, induction of skeletal muscle cell fate has appeared to be a difficult task. While protocols originally relied on overexpression of myogenic transcription factors combined with purification by cell sorting, extensive knowledge of developmental mechanisms (Esteves de Lima and Relaix, 2021) led to protocols recapitulating stepwise signaling cues from embryonic myogenesis (reviewed by Sato, 2020; Caron et al., 2023). In spite of improvements regarding the generation of muscle cells from hiPSC, the degree of maturation of muscle cells resulting from these protocols has remained limited. To fill these shortcomings with *in vitro* muscle cell culture, various stimulation protocols have been implemented such as electrical (Banan Sadeghian et al., 2018; Nagamine et al., 2018; Khodabukus et al., 2019; Nikolić and Aas, 2019; Villanueva et al., 2019; Marš et al., 2021), mechanical (Candiani et al., 2010; Aguilar-Agon et al., 2019; Somers et al., 2019; Moustogiannis et al., 2020; Wang et al., 2020), ultrasound (Abrunhosa et al., 2014; Salgarella et al., 2017) and opto-genetic stimulation (Asano et al., 2015; Asano et al., 2017). This went along with a rising interest in tissue-engineered skeletal muscle models, thus embracing 3D-bioprinting and chemical cues provided by matrix (reviewed by Dessauge et al., 2021; Khodabukus, 2021; Samandari et al., 2022) as well as skeletal muscle organoid generation (Mavrommatis et al., 2023; Shahriyari et al., 2023). Additionally, myogenic maturation features such as sarcomeric striation and spontaneous contraction have been described using sphere-based cultures of myogenic progenitors derived from hiPSC (Hosoyama et al., 2014; Jiwlawat et al., 2017). Even though these methods significantly enhanced the general degree of maturation that myotubes can reach *in vitro*, achieving the formation of neuromuscular synapses similar in function and/or morphology to NMJs remained challenging (reviewed in Luttrell et al., 2021; Lynch et al., 2022; Gazzola and Martinat, 2023; Kim et al., 2023).

Indeed, NMJs are specialized synapses transmitting electrical signals from motor neurons to skeletal muscle fibers to promote muscle contraction via the neurotransmitter, acetylcholine (ACh), that activates nicotinic acetylcholine receptors (nAChR) followed by excitation-contraction coupling (ECC). During embryonic myogenesis, nAChR are pre-patterned in clusters without any neuronal input (Lin et al., 2008). These aneural nAChR were shown to guide motor neuron apposition. Perinatally, the nAChR subunit composition in NMJs switches from γ to ε (Mishina et al., 1986; Witzemann et al., 1987; Witzemann et al., 1989; Charbonnier et al., 2003), nAChR cluster shape changes relying on cytoskeleton remodeling (Dai et al., 2000; Alvarez-Suarez et al., 2021), and subsynaptic nuclei acquire specific transcriptional profiles that contribute to local enrichment of proteins required for NMJ functioning (Sanes and Lichtman, 2001; Schaeffer et al., 2001; Shi et al., 2012; Ohkawara et al., 2023). In existing *in vitro* models, nAChR clustering is usually enhanced by coculturing muscle cells with motor neurons, but only few publications use fully hiPSC-derived models (Bellmann et al., 2019; Andersen et al., 2020; Badu-Mensah et al., 2020; Guo et al., 2020; Lynch et al., 2022). Partially, these studies identified differences in AChR plaque formation as well as contractile and metabolic characteristics between healthy and ALS-derived cell cultures (Lynch et al., 2019; Badu-Mensah et al., 2022). However, myotubes showed a limited differentiation level and the occurrence of nAChR plaques was rare (Badu-Mensah et al., 2020). Other options used co-differentiation of muscle cells and motor neurons in a dish (Mazaleyrat et al., 2020; Urzi et al., 2023) or organoid generation (Faustino Martins et al., 2020; Bombieri et al., 2024).

In this study, we derived hiPSC-derived skeletal muscle cells from control and *SOD1* D90A mutant hiPSC cells and found altered expression of myogenic markers and nAChR-dependent Ca^2+^ responses in mutant myotubes compared to control cells. Moreover, *in vitro* culture conditions were optimized to display enhanced myotube maturation, thus allowing the formation of more complex nAChR clusters in mono- and co-culture conditions with control motor neurons. This revealed intrinsic differences in nAChR cluster morphology between mutant and control myotubes that were reduced by coculture with control motor neurons.

## 2 Materials and methods

### 2.1 hiPSC culture

hiPSC cultures were maintained under feeder-free condition on hESC-qualified Geltrex (Invitrogen, cat. no. A1413302) coated standard 6-well tissue culture plates in mTeSR1 medium (STEMCELL Technologies, cat. no. 85850) with daily medium changes. Once reaching 70%–80% of confluency, hiPSC were routinely passaged with Versene (Invitrogen, cat. no: 15040-066) at a 1:6 ratio. hiPSC and hiPSC-derived cell cultures were cultured at 37°C and 5% CO_2_ in a humidified incubator. Control hiPSC lines (KOLF1.2; 028#1 and 009#3 provided by Philipp Koch, HITBR Hector Institute for Translational Brain Research, Mannheim, Germany) and WC034i-SOD1-D90A (WiCell Research Institute, Madison, WI, United States) were used.

### 2.2 hiPSC skeletal myogenic determination

hiPSC were induced towards skeletal myogenic fate following a previously published protocol (Chal et al., 2016) with minor adjustments. Briefly, 70%–80% confluent hiPSC were treated for 2 h with 10 µM Y-27362 dihydrochloride (ROCKi; Cell Guidance Systems, cat. no. SM02-10) in mTeSR1. hiPSC were washed once with phosphate-buffered saline (PBS) and cells, cultured in 6-well tissue culture plates, were incubated for 5 min with 2 mL TrypLE Express (Invitrogen; cat. no: 12605028). Singularized hiPSC were collected into DMEM/F12 (Capricorn; cat. no: DMEM-12-A) and counted. Cells in suspension were centrifuged at 300 × g for 5 min, and subsequently seeded at a density of 20,000 cells/cm^2^ in mTeSR1 supplemented with 10 µM ROCKi into standard 6-well tissue culture plates coated with hESC-qualified Geltrex. mTeSR medium was refreshed daily. Once 15%–20% confluency was reached, skeletal myogenic targeted determination was induced (D0) based on sequenced media. At D0, hiPSC cultures were switched to DiCL medium containing DMEM/F12 supplemented with 1% Insulin Transferrin Selenium (Invitrogen; cat. no: 41400045), 1% nonessential amino acids (Capricorn; cat. no: NEAA-B), 1% Glutamax (Invitrogen; cat. no: 35050038), 0.2% penicillin/streptomycin (P/S; Capricorn; cat. no: PB-B), 3 µM CHIR99021 (Bio-Techne; cat. no: 4423) and 0.5 µM LDN-193189 (Cell Guidance Systems; cat. no: SM23). At D3, DiCLF medium was applied, and consisted of DiCL medium supplemented with 20 ng/mL recombinant murine FGF-2 (Peprotech; cat. no: 450-33). When reaching D6, cells were switched to DKHIFL medium containing DMEM/F12 supplemented with 15% Knock-out serum replacement (Invitrogen; 10828028), 1% nonessential amino acids, 1% Glutamax, 0.2% P/S, 0.1 mM 2β-mercaptoethanol (Invitrogen; cat. no: 21985), 10 ng/mL recombinant murine HGF (Peprotech; cat. no: 315-23), 2 ng/mL recombinant human IGF-1 (Peprotech; cat. no: 100-11), 20 ng/mL recombinant FGF-2 and 0.5 µM LDN-193189. At d8, DKI medium was applied and consists in DMEM/F12 supplemented with 15% KSR, 1% nonessential amino acids, 1% Glutamax, 0.2% P/S, 0.1 mM 2β-mercaptoethanol and 2 ng/mL recombinant IGF-1. From D0 to D11, medium was refreshed daily. From D12 on, medium was switched to DKHI medium whose composition is similar to DKI medium with addition of 10 ng/mL recombinant HGF. Cells were maintained in DKHI medium until D50 with medium refreshment every other day. Throughout the sequenced media protocol, all media containing knock-out serum replacement were kept away from direct light and stored in light-protected containers. After 50 days of myogenic determination from hiPSC, hiPSC-derived cells were composed of a mixed myogenic population containing myoblasts, myotubes, and satellite-like cells. Myoblasts were purified and subcultured myoblasts were expanded in skeletal muscle cell growth medium (SMCGM; PELOBiotech; cat. no: PB-MH-272-0090) and further cryopreserved in SMCGM supplemented with 10% dimethyl sulfoxide and 10 µM ROCKi.

### 2.3 Terminal differentiation

Myoblasts were seeded in SMCGM onto 96-well µ-Plate (Ibidi; IbiTreat; cat. no: 89626) or 8-well µ-Slide (Ibidi; IbiTreat; cat. no: 80806) previously coated with Geltrex, at a seeding density of 100,000 cells/cm^2^ for control (028#1 line) and *SOD1* D90A lines, and at a seeding density of 150,000 cells/cm^2^ for KOLF1.2 and 009#3 lines. It is important to note that each hiPSC line behaved differently (i.e., proliferation rate, differentiation efficiency), and the seeding density therefore had to be adjusted to each hiPSC line to afford a comparable differentiation index amongst lines. 10 μM ROCKi was added upon seeding, and 24 h later, SMCGM was refreshed. Myoblasts were kept for 6 days in proliferating SMCGM with medium refreshment every other day and were then induced to terminally differentiate by switching to N2-based medium containing DMEM/F12 supplemented with 1% Insulin Transferrin Selenium, 1% N2 (Invitrogen, cat. no: 17502048), 0.2% P/S, 1% Glutamax. Cells were cultured in this medium for 4 days, and medium was refreshed every other day.

### 2.4 Fluo4-mediated Ca^2+^ live-cell imaging

Four days differentiated myotubes were grown in 96-well µ-Plates (Ibidi; IbiTreat; cat. no: 89626) as described in Section 2.3, and incubated for 30 min at + 37 C, 5% CO_2_ with 1 µM Fluo4 calcium indicator (Invitrogen; cat. no: F14201) diluted in N2-based medium supplemented with 10 µM ROCKi. Cells were washed once with N2-based medium and incubated for 20 min at room temperature (RT) additionally to allow the de-esterification of acetoxymethyl moieties of Fluo4 dye. In the condition where cells were pre-treated with α-bungarotoxin (αBGT) before imaging, 3 μg/mL αBGT-AF647 (Invitrogen, cat. no: B35450) was diluted into N2-based medium supplemented with 10 µM ROCKi and incubated at RT for 20 min, before being washed with N2-based medium. Acetylcholine solution (ACh; Tocris; cat. no: 2809) was prepared in N2-based medium supplemented with 10 µM ROCKi. After 30 s of imaging, N2-based medium was added to verify the independency of intracellular calcium transients upon fluid addition into the culture well. After 1 min of imaging, ACh solution was applied on cells at a final concentration of 500 nM. Imaging settings are described in Section 2.7.

### 2.5 Optimized myogenic maturation protocol

Myoblasts were seeded in 96-well µ-Plates (Ibidi; IbiTreat; cat. no: 89626) and left for proliferation for 6 days in SMCGM before switching to differentiation (d0) in N2-based medium as described in 2.3. After 2 days of differentiation (d2), medium was partially removed leaving 10 μL of residual medium in the well. 85 µL/well of heSC-qualified Matrigel (Corning; cat. no: 354277) diluted into N2-based medium at a ratio of 1:1 was shortly applied to cells, and subsequently incubated for 45 min at +37°C, 5% CO_2_. 350 μL of N2-based medium was added to fill up the well. At d3, medium was switched to maturating medium, with a composition adapted from Hörner et al. (2021). Its base medium contained neurobasal medium (Invitrogen; cat. no: 21103049) and DMEM/F12 medium without HEPES (Invitrogen; cat. no: 10565018) in a 1:1 ratio, 1% P/S, 1% GlutaMAX, 0.5X NeuroCult SM1 Supplement (STEMCELL Technologies; cat. no: 05711), 0.5X N2 Supplement, and supplemented with 0.1 mM ascorbic acid (Carl Roth; cat. no: 3525.1), 3 µM CHIR99021, 2 µM DMH1 (Bio-Techne; cat. no: 4126), and 2 µM SB431542 (Bio-Techne; cat. no: 1614). This base medium was supplemented with 0.1 µM purmorphamine (Cell Guidance Systems; cat. no: SM30), 0.5 µM all-trans retinoic acid (STEMCELL Technologies; cat. no: 72264), 10 ng/mL recombinant human BDNF (Cell Guidance Systems; cat. no: GFH1AF), 10 ng/mL recombinant human GDNF (Cell Guidance Systems; cat. no: GFH2AF), and 10 ng/mL recombinant human IGF-1. While switching to maturating medium at d3, hiPSC-derived motor neurons (iMN) were eventually added to the myogenic culture. For this purpose, iMN were determined from hiPSC and differentiated as described by Hörner et al. (2021). Briefly, at day 12, differentiating iMN were split into suspension culture. From this step on, cells progressively aggregated into neurospheres. Medium was changed every other day for 1 week by carefully replenishing 75% of medium volume in each well. Neurospheres were seeded onto maturating myotubes at d3 of their differentiation. Maturating medium was refreshed every other day by carefully replenishing 50% of volume in each well until d8. This medium was carefully kept away from light.

### 2.6 α-bungarotoxin sequential staining

Maturing myotubes were obtained as described in Section 2.5. Once myotubes reached 7 days of differentiation, αBGT-AF488 [Invitrogen; cat. no: B13422; (5 μg/mL)] diluted in maturating medium supplemented with 10 µM ROCKi was incubated with living cells for 15 min at + 37°C, 5% CO_2_. Cells were washed with maturating medium and placed back overnight at + 37°C and 5% CO_2_. 24 h later, αBGT-AF647 [Invitrogen, cat. no: B35450; (5 μg/mL)] diluted in maturating medium supplemented with 10 µM ROCKi was incubated for 15 min at + 37°C, 5% CO_2_. Cells were fixed with 4% PFA for 30 min at RT. Samples were washed three times for 5 min with PBS at RT. Imaging was performed as described in Section 2.7.

### 2.7 Immunofluorescence staining and confocal microscopy

Cultures were fixed with 4% paraformaldehyde at room temperature (RT) for 15 min for d4 differentiated myotubes and for 30 min for d8 matured myotubes and washed three times with PBS before being further processed with immunostaining. Samples were permeabilized with 1x Tris-Buffered Saline (TBS) buffer supplemented with 0.1% Tween-20, three times for 3 min at RT. Cells were incubated in blocking solution containing 1x TBS supplemented with 1% fetal bovine serum and 0.1% Triton-X100, for 30 min at RT. Primary antibodies diluted in blocking solution were incubated overnight at + 4°C. After three PBS washes, secondary antibodies and dyes were diluted into blocking solution and incubated for 3 h at RT. Samples were washed three times for 5 min at RT with permeabilization buffer and one last time with PBS before being imaged. Primary antibodies and dye concentrations were as follows: anti-Myogenin (MyoG; LSBio; cat. no: LS-C334865; dilution: 1/400), anti-Myosin Heavy Chain 1 (MYH1; DSHB; cat. no: MF20-c; dilution: 1/800), anti-α-actinin (Invitrogen; cat. no: MA1-22863; dilution: 1/400), anti-vesicular acetylcholine transporter (vAChT; Synaptic Systems; cat. no: 139 103; dilution: 1/400), 4’,6-Diamidino-2-Phenylindole (DAPI; Roche; cat. no: 10236276001; 1 mg/mL; dilution 1:1000); αBGT-AF488 (Invitrogen; cat. no: B13422; dilution: 1/500), αBGT-AF647 (Invitrogen; cat. no: B35450; dilution: 1/500), Donkey α-mouse AF-488 (Invitrogen; cat. no: A21202; dilution 1:1000), Donkey α-rabbit AF-555 (Invitrogen; cat. no: A32794; dilution 1:1000), Donkey α-mouse AF-647 (Invitrogen; cat. no: A31571; dilution 1:1000). Imaging was performed using an inverted Leica TCS SP8 confocal microscope (Leica Microsystems) with HC PL APO 20x/0.75 IMM CORR CS2, and 405, 488, 561, and 633 nm lasers and Leica Application Suite X software (version 3.5.7.23225). Mainly, images were acquired as single focal plane images, with a resolution corresponding to a pixel size of 0.28 μm × 0.28 μm. However, for staining designated for αBGT abundancy quantification, z-stacks were imaged with 1 µm z-steps size corresponding to a pixel size of 0.56 μm × 0.56 μm and a voxel depth of 4 μm^3^. Calcium transient recording was performed with HC PL APO 20x/0.75 IMM CORR CS2 objective, 488 nm lasers, with 1024 × 1024 pixels resolution corresponding to a pixel size of 0.56 μm × 0.56 μm and 2 s time interval. Images acquired for nAChR clusters morphological analysis and αBGT-AF488 positive puncta quantification were performed with a HC PL APO 63 × /1,40 OIL CS2 objective, and 488 and 633 nm lasers. Images were acquired sequentially. Z-stacks were acquired to capture the entirety of the imaged clusters. A z-step size of 0.3 µm was set and images were acquired with a resolution corresponding to a pixel size of 0.045 μm × 0.045 μm and a voxel depth of 0.29 μm^3^. Randomized areas were imaged for all experiments.

### 2.8 Image analysis and processing

Quantification of DAPI-stained and MyoG-positive nuclei was achieved by using Cellpose (version 2.2.2) (Stringer et al., 2021). Before automated quantification analysis, a dataset of five images for each staining was used for testing and algorithm training. Differentiation index was achieved in ImageJ software by manually quantifying the number of nuclei within MYH1-positive cells. For MyoG and MYH1 mean fluorescence intensity quantification, MyoG-related regions of interest (ROI) generated in Cellpose and MYH1-related ROIs created with thresholding in ImageJ were used. To define the threshold, background fluorescence intensity was measured in five regions per sum-z-projected image, and the average and standard deviation were calculated. Background average fluorescence intensity + 2× (Standard deviation) was defined as threshold, and cell displaying greater values in sum-z-projected images than the threshold was counted as positive for the marker; otherwise, the cell was counted as negative. Cell debris were excluded by deleting ROIs with an area smaller than 500 mm^2^ and a circularity from 0.3 to 1.0. Ca^2+^ responses of myotubes upon ACh stimulation were analyzed with ImageJ software. First, myotubes were manually segmented. The mean fluorescence values within the segmented areas were determined over time and normalized to the corresponding mean signals 6 s before stimulation (F_0_). The change in fluorescence ΔF/F_0_ = (F − F_0_)/F was plotted as a function of time. To obtain muscle cell area quantifications, images of myogenic cultures stained for myogenic markers (MYH1 or α-actinin) were processed in ImageJ software with median filtering with a radius of two pixels. The previously described thresholding method was used to segment MYH1- and α-actinin-positive cells. ROIs were obtained from binary masks. Cell debris were first removed by deleting ROIs with an area smaller than 500 μm^2^ and a circularity from 0.3 to 1.0. Subsequently, remaining ROIs were segmented, allowing the quantification of total muscle cell area. Myonuclear domain area was calculated by the following formula: total muscle cell area/total number of myonuclei. For αBGT-AF488 positive puncta quantification, z-stack images were cropped to position nAChR clusters at the center of a 40 µm muscle cell length. By increasing the contrast of αBGT-AF647 channel, the outline of myotubes became visible, allowing manual segmentation. The quantification was performed on the stack covering the entire thickness of the cluster. The counting of αBGT-AF488-positive puncta was performed manually in ImageJ software. Quantified values were normalized to myotube volume. For segmentation and morphological analysis of nAChR clusters, maximum-z-projections were used. Single nAChR clusters were cropped from images as rectangular ROIs, containing only signals from one cluster for each ROI. Hereby, only “*en face*” clusters were chosen, while clusters imaged from a side view were excluded to prevent distortion of shape parameter measurement. ROI masks were created by manual thresholding and contrasting αBGT-647 signal to measure shape parameters including cluster area, perimeter, and solidity were measured.

Image preprocessing for Supplementary Figure S1; Supplementary Video S6 were performed using ImageJ, specifically adjusting brightness and contrast across all channels for optimal visualization. To accentuate the distribution of vesicular acetylcholine transporter (vAChT) signals, the relevant channel underwent additional processing. This involved generating a binary image through Otsu thresholding, followed by 3D morphological opening and dilation operations, each with a radius of one voxel in three dimensions. Connected structures were identified using the connected components labeling algorithm from the MorpholibJ library. Subsequent to this labeling, size filtering was applied to exclude structures smaller than 1,000 voxels or larger than 115,000 voxels. An additional dilation step with a radius of two pixels was then performed, and a new binary mask was created via Otsu thresholding. This mask was used to selectively remove vAChT signal outside of the identified binary labels. Videos were subsequently generated using the visualization software, napari (Ahlers et al., 2023), in conjunction with its dedicated animation plugin. This combination facilitated dynamic visual representations of the data.

### 2.9 Data processing and statistics

**FIGURE 1:**
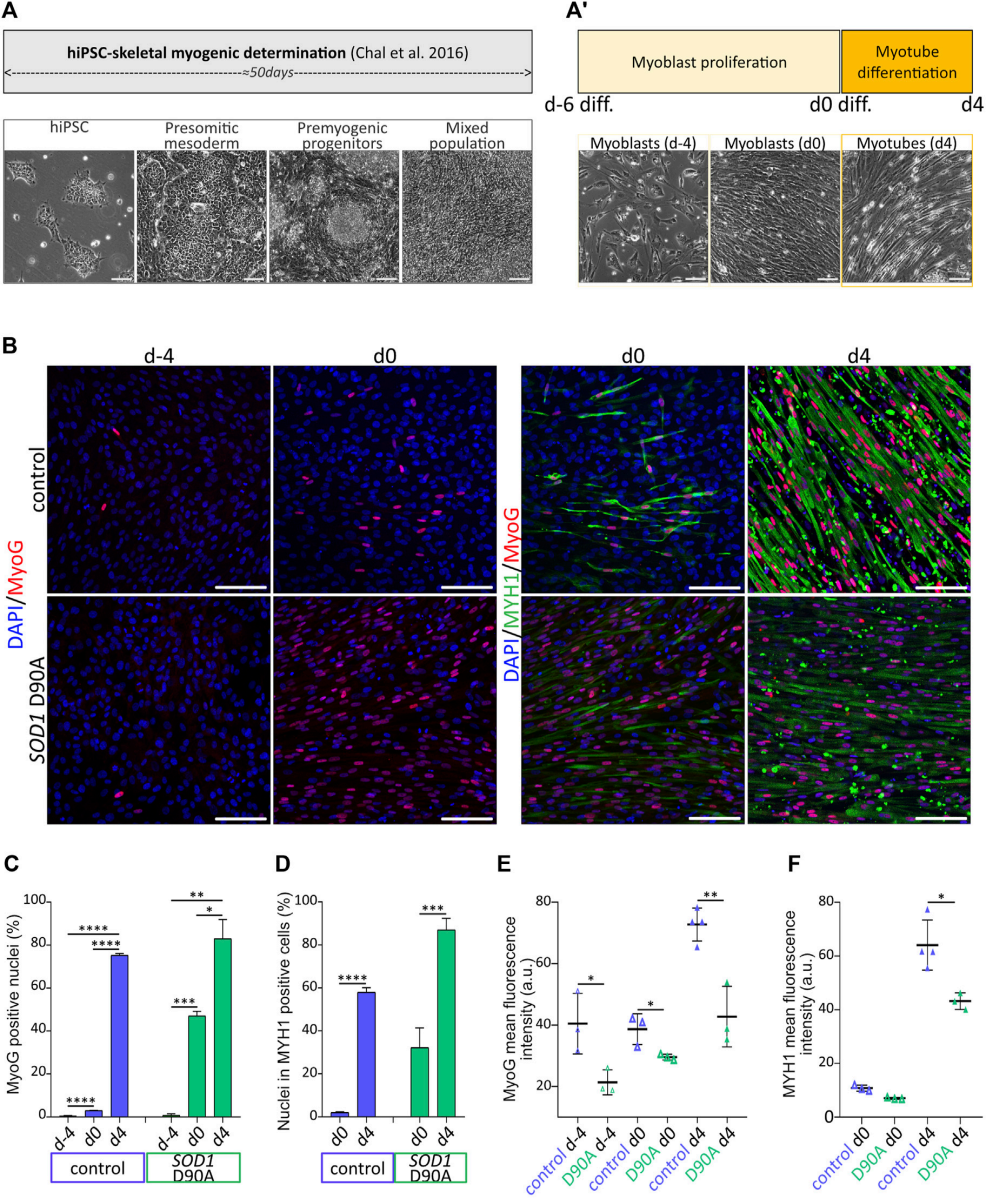
Evolution of marker protein expression along terminal myogenesis indicates differentiation of skeletal myotubes from hiPSC*.* hiPSC were determined and further differentiated for up to 60 days. **(A-A′)** Upper panels: schematic protocol timeline for hiPSC determination towards skeletal myogenic fate **(A)** and hiPSC-derived myoblast terminal differentiation **(A′)**. Lower panels: representative brightfield images of cell populations at various determination and differentiation timepoints. Presomitic mesoderm cells, premyogenic progenitors and mixed cell population consisting of myoblasts and myotubes were imaged at d-45, d-40 and d-22, respectively. hiPSC-derived myoblasts were obtained after 50 days of hiPSC determination **(A)** and were further induced to terminally differentiate into myotubes after 6 days of proliferation and 4 days of differentiation in N2-based medium **(A′)**. Scale bars, 100 µm. **(B–F)** hiPSC-derived myoblasts were differentiated according to the protocol shown in **A-A′**, fixed at different timepoints, and immunostained for myogenic markers. Skeletal muscle cells were derived from control and *SOD1* D90A mutant hiPSC lines. **(B)** Representative confocal images of samples immunostained for myogenin (MyoG) and myosin heavy chain (MYH1) at timepoints as indicated. Scale bars, 100 µm. **(C,D)** Quantification of the percentage of MyoG positive nuclei **(C)** and of percentage of nuclei within MYH1 positive cells (differentiation index; D) as a function of differentiation time. Graphs depict mean ± SD. **(E,F)** Quantification of MyoG **(E)** and MYH1 **(F)** mean fluorescence intensity of positive cells. At least three biological replicates were analyzed per condition. *p < 0.05, **p < 0.01, ***p < 0.001, ****p < 0.0001.

**FIGURE 2:**
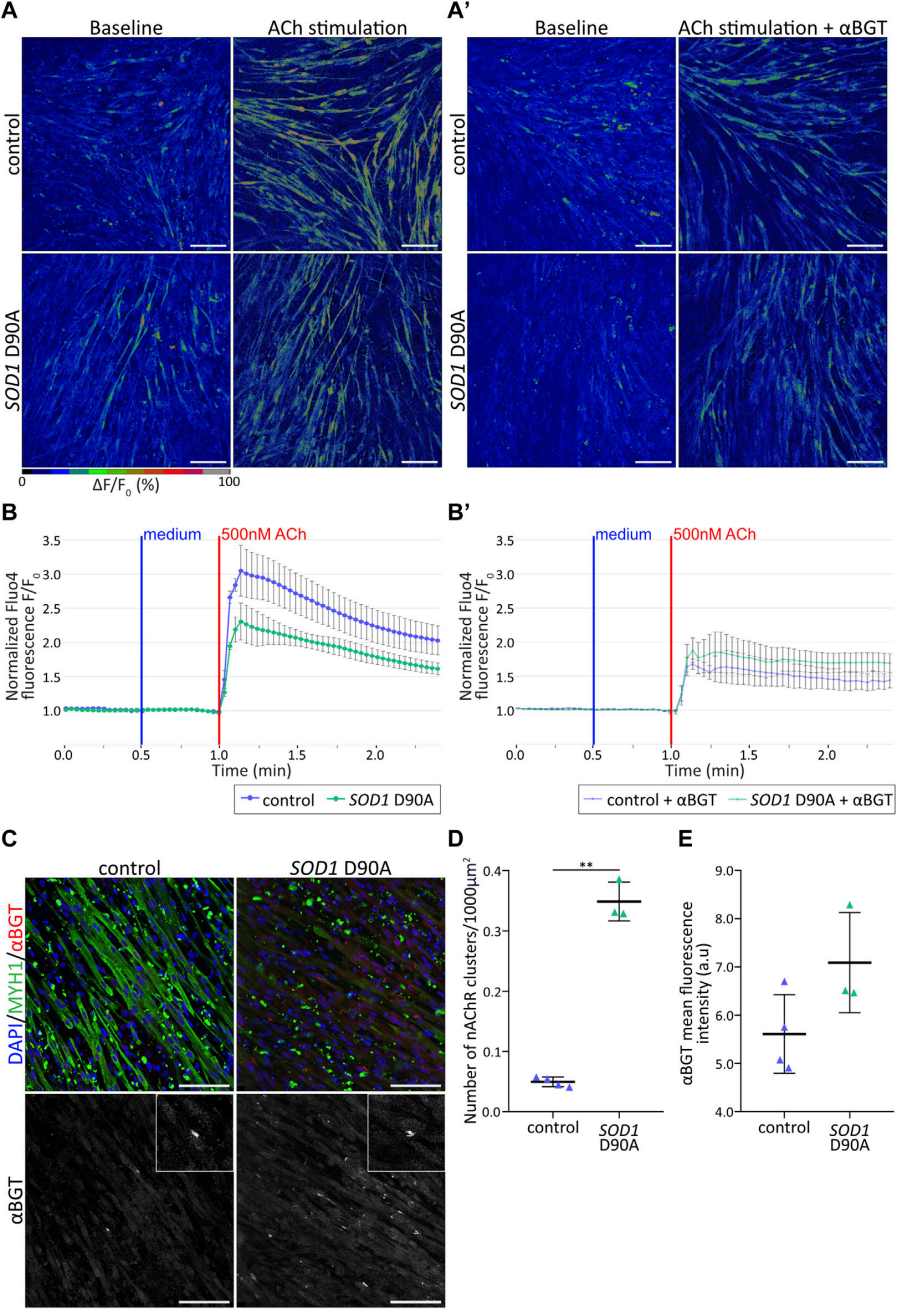
Control and SOD1 D90A mutant hiPSC-derived myotubes show differential nAChR-dependent Ca2+ responses*.* **(A,B′)** hiPSC-derived myotube cultures differentiated for 50 + 4 days were subjected to Fluo4-mediated Ca^2+^ imaging in the presence of acetylcholine [ACh; (500 nM0)] ± α-bungarotoxin [αBGT; (3 μg/mL)]. **(A)** Representative confocal pseudocolored images for all conditions at baseline (left panels) and peak upon ACh (right panels). Myotubes were stimulated with ACh either directly **(A)** or after pre-treatment with αBGT **(A′)**. The pseudocolor scale bar shows the color distribution corresponding to Fluo4 fluorescence ratios. Blue and green-red cues indicate low and high values of Fluo4 fluorescence, respectively. Scale bars, 100 µm. **(B-B′)** ΔF/F_0_ Fluo4 kinetics for control and *SOD1* D90A mutant myotubes upon ACh stimulation, without **(B)** and with **(B′)** αBGT pre-treatment. Fluo4 fluorescence was normalized to corresponding baseline values. Curves depict mean ± SE of at least three biological replicates. **(C)** Representative confocal images of control and mutant myotubes showing fluorescence signals of nuclei (blue), αBGT-stained nAChR clusters (red), and MYH1 (green). Inserts in right panels depict higher zooms of nAChR clusters. Scale bars, 100 µm. **(D,E)** Quantification of nAChR clusters per 1000 µm^2^ of myotubes **(D)** and of αBGT integrated density **(E)** in control and mutant conditions. n = 3 independent experiments. **p < 0.01.

**FIGURE 3:**
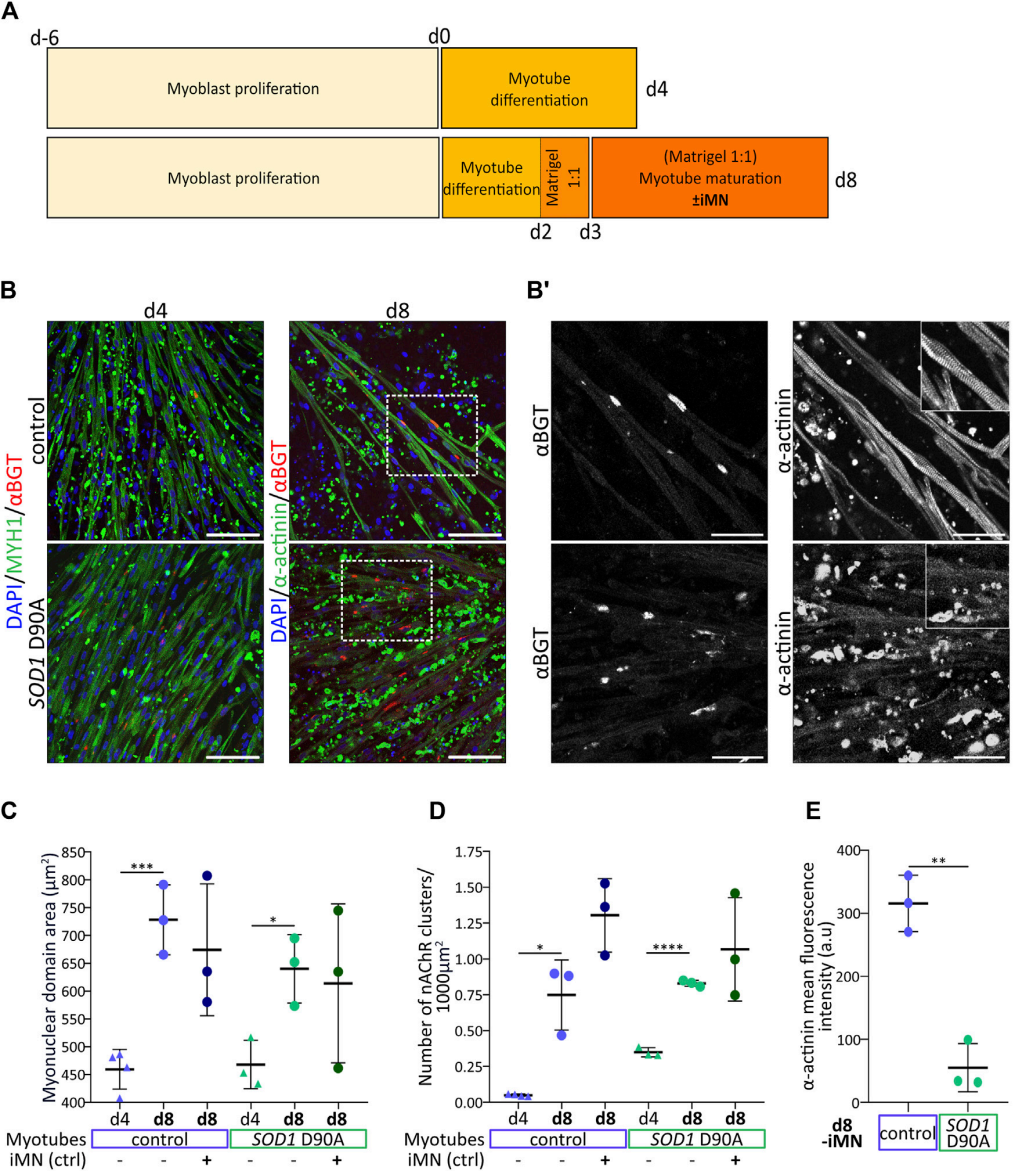
Optimized culture conditions enhance myotube maturation and highlight a lack of marker expression and sarcomeric organization in SOD1 D90A myotubes. Myoblasts were expanded for 6 days (d-6 to d0) and then further differentiated for 4 or 8 days (d0 to d4/d8). In some conditions, maturating myotubes were cocultured with control iMN, as indicated. **(A)** Comparison of protocols and timelines leading to newly differentiated myotubes (d4-protocol) and more mature myotubes (d8-protocol). **(B)** Representative confocal fluorescence images of control and *SOD1* D90A mutant myotubes as obtained with the d4 and d8 protocols (indicated) in the absence of iMN. Fluorescence signals show nuclei (blue), αBGT-stained nAChR clusters (red), and MYH1 or α-actinin (green). Scale bars, 100 µm. Dashed rectangles outline higher magnification areas shown in B’. **(B′)** Gray-scaled zooms of αBGT and α-actinin staining in d8-differentiated control and mutant myotubes are shown. Scale bars, 50 µm. Inserts in right panels depict higher zooms of individual myotube striations. **(C–E)** Quantification of myonuclear domain area **(C)**, nAChR clusters per 1000 µm^2^ of muscle cell area **(D)** and α-actinin mean fluorescence intensity **(E)** comparing d4 and d8 myotubes in mono- (-iMN) and co-culture with control iMN ( + iMN). Graphs depict mean ± SD and were generated from data obtained from at least three individual experiments. *p < 0.05, **p < 0.01, ***p < 0.001, ****p < 0.0001.

**FIGURE 4:**
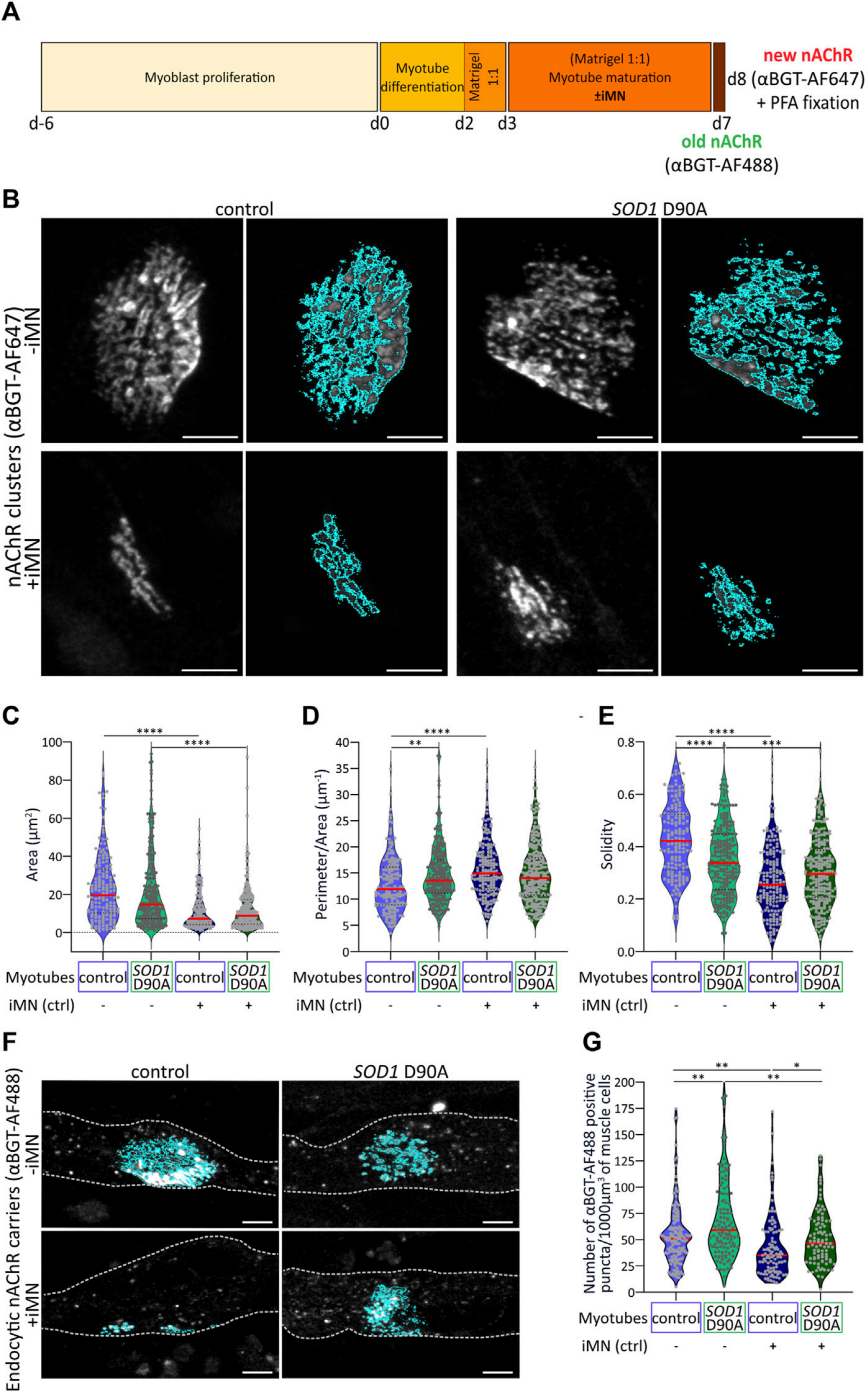
Differences in morphological parameters and stability of nAChR clusters between control and SOD1 D90A mutant myotubes are reduced upon coculture with control iMN*.* Myoblasts were expanded for 6 days (d-6 to d0) and then further differentiated for 8 days (d0 to d8). In some conditions, maturating myotubes were cocultured with control iMN, as indicated. At d7 and d8, αBGT-AF488 and αBGT-AF647, respectively, were added to live cultures for 15 min. Then, samples were washed, fixed, and visualized with confocal microscopy. **(A)** Schematic protocol timeline to assess nAChR cluster turnover through a sequential αBGT living cells staining. **(B)** Gray-scaled pictures of αBGT-AF647-stained nAChR clusters (left panels) and their corresponding segmentation masks obtained by thresholding-based segmentation in ImageJ for all conditions (right panels). Scale bars, 5 µm. **(C–E)** Quantification of cluster area **(C)**, perimeter/area **(D)**, and solidity **(E)**. Red lines, mean values for each condition. **(F)** Representative images of d8 control and *SOD1* D90A myotubes cultured as mono- (upper panel; -iMN) or iMN co-cultures (lower panel; + iMN). Dotted lines outline myotubes. Scale bars, 5 µm. **(G)** Quantification of αBGT-AF488 positive puncta per 1,000 μm^3^ of muscle cell volume. Red lines, mean values for each condition. At least 50 nAChR clusters were analyzed per condition, and three independent experiments were performed. *p < 0.05, **p < 0.01, ***p < 0.001, ****p < 0.0001.

Graphic representation of data was achieved with GraphPad Prism (version 8.0.1). Using the same software, statistical tests were performed as follows. Shapiro-Wilk test for normal distribution and F-test for homoscedasticity were performed. Student’s *t*-tests were used to statistically compare results shown in Figures 1, 2, 3. When not pertinent, Student’s *t*-tests with Welch’s correction were applied instead. Statistical analyses shown in Figure 4 were performed by using ANOVA with Tukey’s multiple comparisons tests if applicable, rank-based nonparametric Kruskal Wallis tests were performed otherwise. *p*-value smaller than 0.05 was considered significant and reported as *p < 0.05, **p < 0.01, ***p < 0.001, ****p < 0.0001. The number of independent experiments performed is indicated in the figure legends. Figures were prepared in Affinity Designer (version 1.10.5.1342).

## 3 Results

### 3.1 Skeletal myotubes can be efficiently differentiated from hiPSC

Determination and differentiation of hiPSC towards skeletal muscle fate were achieved by applying a sequence of differentiation factors on hiPSC, as described previously (Chal et al., 2016), but with slight modifications. Indeed, while the initial 50 days of the protocol reaching the myoblast stage (Figure 1A) followed the earlier accounts (Chal et al., 2016), we optimized the process of myoblast proliferation and myotube differentiation by adapting i) the myoblast seeding density prior to terminal differentiation induction as well as ii) the duration of myoblast proliferation and myotube differentiation. Regarding the control 28#1 and the *SOD1* D90A lines, 100,000 cells/cm^2^ were seeded, while for the control lines KOLF1.2 and 009#3 (Supplementary Figure S1), 150,000 cells/cm^2^ were seeded. KOLF1.2 and 009#3 displayed reduced mitotic frequency compared to 28#1 and *SOD1* D90A lines. KOLF1.2 and 009#3 lines required additionally a denser cell layer prior to differentiation initiation to efficiently and reproducibly shape myotubes after 4 days of differentiation. By increasing the seeding density for the KOLF1.2 and 009#3 lines, reduced proliferation rate and differentiation efficiency were counterbalanced and allowed the obtention of myotubes with comparable phenotypes amongst 28#1, *SOD1* D90A, KOLF1.2 and 009#3 cell lines after 4 days of differentiation (data not shown). In total, this process lasted 6 days for myoblast proliferation (days −6 to 0) and another 4 days for myotube differentiation (days 0–4) (Figure 1A’). Differentiation batches derived from 28#1 (hereafter named control) and *SOD1* D90A cells were directly compared. From days d-6 to d4, the expression of the myogenic marker proteins, MyoG and MYH1, gradually increased in both lines (Figures 1B–D). Indeed, on d-4 only a few myoblasts were observed in both lines, and MyoG was barely expressed. On d0, MyoG expression was still low in control (3.05% ± 0.09%; mean ± SD), while *SOD1* D90A cells already showed 47.09% ± 2.12% of total cells (mean ± SD) positive for MyoG. By d4, both lines exhibited a similar degree of MyoG-positive cells, although the intensity of MyoG-immunofluorescence signals per cell was higher in control than in *SOD1* D90A cells (Figures 1B, E). As visible from the MYH1-staining data, myoblasts from both lines started to fuse with each other around d0 and formed elongated myotubes (see MYH1 staining in Figure 1B, d0). This process was seen throughout the entire culture by d4 (Figure 1B, see MYH1 panels). Accordingly, a strong increase of MYH1 expression was observed during this time period. Indeed, while at d0 2.01% ± 0.46% and 32.21% ± 9.24% (both mean ± SD) of nuclei were found in MYH1-positive control and *SOD1* D90A myotubes, respectively, these numbers increased at d4 to 58.04% ± 2.07% and 87.07% ± 5.28% (mean ± SD), respectively (Figures 1B–D). Similar as for MyoG fluorescence intensity (Figures 1B, E), the MYH1 fluorescence intensity per cell was higher in control myoblasts and myotubes as compared to *SOD1* D90A cells (Figures 1B–F). In summary, the rise of expression of both myogenic markers throughout the protocol indicated an efficient myogenic process for both cell lines, however, with differences in time course and protein expression height between control and *SOD1* D90A cells.

### 3.2 *SOD1* D90A mutant hiPSC-derived myotubes show altered nAChR-dependent Ca^2+^ responses compared to control myotubes

To characterize the myotube differentiation from a functional point of view, their response to the natural agonist, ACh, was tested. Physiologically, ACh activates nAChR at the NMJ, leading in sequence to the formation of an endplate potential and an action potential, the release of Ca^2+^ from internal stores, and muscle contraction (ECC) (for recent review, see Salvage et al., 2023). To investigate nAChR-dependent Ca^2+^ responses in control and *SOD1* D90A myotubes, d4-myotubes were incubated with the intensity-based Ca^2+^ indicator, Fluo4 (Gee et al., 2000). In both cell lines, addition of 500 nM ACh led to immediate and robust cytoplasmic [Ca^2+^] transients (Figures 2A, B; Supplementary Videos S1–S2). Quantitative analysis showed that the transient peaks tended to be smaller in *SOD1* D90A compared to control myotubes determined from three lines that showed comparable response (Figure 2B; Supplementary Figure S1). To address, if the [Ca^2+^] transients upon ACh stimulation were due to nAChR activation, the selective nAChR antagonist, αBGT, was incubated prior to ACh stimulation. In control myotubes, αBGT led to a significant drop of 43.6% of the transient peak compared to the condition lacking the inhibitor. Conversely, in mutant myotubes αBGT preincubation led only to a 12.4% reduction (Figures 2A’, B’; Supplementary Figure S1; Supplementary Videos S3–S4). To verify whether the greater cytoplasmic [Ca^2+^] transients in control myotubes could be related to the nAChR total amount or the nAChR cluster abundancy, d4-myotubes from both cell lines were stained for nuclei (DAPI), MYH1, and nAChR (αBGT) and imaged with confocal microscopy (Figure 2C). Quantification of the number of nAChR clusters per muscle cell area and the integrated αBGT-fluorescence density showed a significantly higher number of nAChR clusters per muscle cell area in *SOD1* D90A myotubes compared to control myotubes (Figure 2D), associated with a trend for increased integrated αBGT-fluorescence density (Figure 2E). Taken together, these results demonstrated a slightly reduced Ca^2+^ response upon ACh stimulation in *SOD1* D90A myotubes compared to control myotubes and suggest that this occurred independent of the amount of nAChR or of their clustering.

### 3.3 *SOD1* D90A myotubes lack sarcomeric striation upon enhanced myotube maturation

While protocols to derive skeletal muscle cells from hiPSC are constantly refined and have achieved high yields of myoblasts (reviewed in Sato, 2020), the terminal differentiation into well-matured myotubes has remained under-explored. To support myotube maturation without the need of using complex stimulatory or mechanical devices, we worked on optimizing the *in vitro* culture conditions. In brief, at difference to the original myotube differentiation protocol (termed protocol d4 in Figure 3A), myotubes differentiated for 2 days were covered by a Matrigel layer and then matured in a growth-factor-supplemented medium (see Materials and Methods section) in the presence or absence of control motor neurons (iMN) (Figure 3A, protocol d8). Protocol efficiency was addressed by confronting myotube maturation parameters of d4-myotubes (raised with protocol d4, Figure 3A) and d8-myotubes (cultured according to protocol d8, Figure 3A). To that end, myotubes were stained for nuclei (DAPI), MYH1 or sarcomeric α-actinin (α-actinin), and nAChR (αBGT), followed by confocal microscopy and quantitative analysis of the myonuclear domain area (MND) and the density of nAChR clusters. Qualitatively, d8-myotubes from both cell lines appeared to be longer, wider, and with larger nAChR clusters as compared to d4-myotubes (Figure 3B). A comparison of control and *SOD1* D90A d8-myotubes showed similar amounts of nAChR clusters (Figure 3B’, left panels). However, while in most control d8-myotubes the α-actinin staining showed extensive striations (Figure 3B, upper right panels, Supplementary Video S5), these were largely absent in the *SOD1* D90A d8-myotubes (Figure 3B’, lower right panels). Quantitative analysis revealed that d8-myotubes in monoculture of both, control and *SOD1* D90A cells, exhibited significantly higher myonuclear domain area compared to d4-myotubes (Figure 3C). Upon coculture with iMN, the d8-myotube values remained essentially unaltered (Figure 3C). As another maturation parameter, the density of nAChR clusters was determined. For both cell lines, nAChR cluster density significantly increased from d4-myotubes to d8-monocultures (Figure 3D). As a trend, the presence of iMN further augmented the nAChR density in control and *SOD1* D90A d8-myotubes (Figure 3D), but the effect was not statistically significant. Similar to d4-*SOD1* D90A myotubes showing reduced differentiation marker protein MYH1 (Figures 1B–F), d8-*SOD1* D90A myotubes displayed significantly lower α-actinin expression compared to control d8-myotubes (Figure 3E). Of note, in the coculture setup, iMN established close contacts with maturating myotubes (Supplementary Figure S2; Supplementary Video S6). In summary, while contraction-induced muscle-cell death needs to be further addressed, these results revealed differences between control and *SOD1* D90A myotubes with respect to sarcomere formation, as illustrated by the absence of α-actinin striation pattern and the reduced fluorescence intensity of this staining.

### 3.4 Coculture with iMN consolidates nAChR clusters and reduces morphological differences between control and *SOD1* D90A clusters

Although a quantitative assessment showed the similar occurrence of nAChR clusters in d8 control and *SOD1* D90A myotubes (Figures 3B’–D), a morphometric analysis of nAChR clusters and endocytic carriers was performed to identify more subtle differences between both cell lines. To differentiate between nAChR located in endocytic vesicles and clusters on the cell surface, a sequential labeling with two differently fluorescent αBGT species (coupled to either AlexaFluor488 or AlexaFluor647) was performed (Figure 4A), similar to previous *in vivo* experiments (Akaaboune et al., 1999; Röder et al., 2010; Strack et al., 2011). In brief, myotubes were matured according to the d8-protocol in the presence or absence of control iMN. On d7, surface-exposed nAChR were pulse-labeled with αBGT-AF488 (old nAChR). 24 h later, new nAChR were marked with αBGT-AF647 (new nAChR) and then samples were fixed. First, we investigated whether nAChR clusters exhibited morphological differences between control and *SOD1* D90A myotubes in the absence of iMN. As a trend, nAChR clusters forming on mutant myotubes were slightly smaller than those in control cells, but the difference was not significant (Figures 4B, C). However, compared to control cells, mutant myotubes showed increased nAChR cluster perimeter/area (Figures 4B–D) and a decreased solidity (Figures 4B–E). Next, the effects of adding control iMN on nAChR cluster morphology were adding control iMN on nAChR cluster morphology were addressed. Essentially, this led to marked alterations of cluster morphology in both cell lines and reduced all differences observed between control and *SOD1* D90A myotubes in the absence of iMN. In detail, the addition of iMN decreased nAChR cluster area by 50.9% and, 44.1% in control and *SOD1* D90A myotubes, respectively, compared to their corresponding monocultures (Figures 4B, C). Further, in the presence of iMN, perimeter/area of nAChR clusters was increased and solidity was decreased in control myotubes, while in *SOD1* D90A myotubes only the solidity of nAChR clusters was significantly modified (Figures 4B–D, E). This reduced the discrepancies regarding nAChR cluster morphology between both cell types as observed in the absence of iMN. Finally, to investigate whether the alterations in nAChR cluster morphology might be reflected by nAChR trafficking and decay, the number of endocytic nAChR puncta was determined under all conditions. Therefore, nAChR clusters were identified in the αBGT-AF647 images (see outlines in Figure 4F) and then, αBGT-AF488 positive puncta were counted. In the absence of iMN, the amount of nAChR carriers per myotube cell volume was higher in *SOD1* D90A compared to control cells (Figures 4F–G). For both cell lines, the number of αBGT-AF488 positive puncta significantly decreased in the presence of iMN, suggesting a consolidation of clusters under this condition (Figures 4F, G). However, this did not abut the statistical difference between control and *SOD1* D90A myotubes. Altogether, these data demonstrated intrinsic differences between control and *SOD1* D90A myotubes concerning nAChR cluster morphology and endocytic carriers, which were partially reduced by the presence of iMN.

## 4 Discussion

### 4.1 Tuning of chemical and mechanical culture parameters enhances myotube maturation and nAChR cluster formation

This study implemented a protocol to obtain mature myotubes from hiPSC. Its originality relies on a specific maturation medium with key myogenic factors and the provision of matrix-based physical support. The protocol was devised to allow muscle monoculture as well as their coculture with hiPSC-derived motor neurons, supporting their intercellular crosstalk. Thus, to improve myotube maturation, the initial 4-day protocol of myotube differentiation (Figure 1A’) was prolonged to 8 days of culture in specific media, including a 2-day myotube pre-differentiation step to increase myoblast fusion initiation and a 6-day maturation period during which myotubes were covered by a Matrigel layer (Figure 3A). Compared to the previous protocol, this led to significantly improved expression of differentiation markers and an increase of nAChR cluster density (Figures 3B–D). We think that the combination of Matrigel application and the specific medium supplementation were key to efficient hiPSC-derived myotube maturation. Matrigel is a solubilized basement membrane matrix secreted from Engelbreth-Holm-Swarm mouse sarcoma cells, which resembles the basement membrane found in muscle native tissue (Sanes, 2003; Kleinman and Martin, 2005). Indeed, while main components of Matrigel are laminin, collagen IV, and proteoglycans, the native muscle extracellular matrix environment is composed of laminin, collagen I, collagen IV, elastin, and proteoglycans (Csapo et al., 2020). The importance of the skeletal muscle extracellular matrix on development and muscle maintenance (Thorsteinsdóttir et al., 2011; Csapo et al., 2020; Zhang et al., 2021) as well as the benefit of using Matrigel for skeletal muscle cell culture *in vitro* have been reported (Lyles et al., 1992; Grefte et al., 2012). In addition to laminins, which play a key role in nAChR maturation (Nishimune et al., 2008; Chand et al., 2017), Matrigel provides mechanical support and a near-physiological stiffness. Physiologically, Young modulus in skeletal muscles varies according to the myogenic state and ranges from 11.5 to 45.3 kPa (Collinsworth et al., 2002). Stiffer substrates were found to support myoblast proliferation, while softer substrates ranging from 13 kPa to 20 kPa were more suited for differentiation (Boontheekul et al., 2007; Romanazzo et al., 2012; Lacraz et al., 2015; van Santen et al., 2022). These results highlight the unsuitability of commonly used culture dishes to differentiate muscle cells due to their supra-physiological stiffness in the MPa range (Engler et al., 2004). A multitude of signaling pathways can be triggered by mechanical cues, sensed at the sarcolemma, conveyed by the cytoskeleton throughout the cytoplasm and transmitted to the nucleus via the linker of nucleoskeleton and cytoskeleton complex (Nguyen et al., 2024; reviewed in Olsen et al., 2019; Iyer et al., 2021; Jabre et al., 2021; van Ingen and Kirby, 2021; Zhang et al., 2023). Thus, mechanical cues eventually affect key processes of muscle maturation (e.g., myofibrillogenesis) (Jorgenson et al., 2024).

Apart from Matrigel, our maturation protocol also included a carefully designed media composition to enhance myotube maturation, sustain motor neuron survival, and facilitate neuromuscular crosstalk. The factor cocktail was chosen to trigger pathways relevant for both cell types. On the one hand, ascorbic acid (Duran et al., 2019; Diao et al., 2021), ALK5 inhibitor SB431542 (Watt et al., 2010), Hedgehog/Smoothened agonist purmorphamine (Kahane et al., 2013; Teixeira et al., 2018), retinoic acid (Hamade et al., 2006; Ryan et al., 2012; Lamarche et al., 2015; Thulabandu et al., 2022), and IGF-1 (Yoshida and Delafontaine, 2020) were used for their positive influence on motor neuron maturation and survival, and myotube differentiation. On the other hand, the GSK-3 inhibitor and Wnt activator, CHIR99021 (Vertino et al., 2005; van Amerongen and Berns, 2006; Henriquez et al., 2008; Jing et al., 2009; Cisternas et al., 2014; Girardi and Le Grand, 2018), brain-derived neurotrophic factor (BDNF) (Gonzalez et al., 1999; Wells et al., 1999; Clow and Jasmin, 2010; Kulakowski et al., 2011; Je et al., 2013; Rentería et al., 2022), and glial cell line derived neurotrophic factor (GDNF) (Nguyen et al., 1998; Keller-Peck et al., 2001; Zwick et al., 2001; Wang et al., 2002; Stanga et al., 2016; Stanga et al., 2020) were included to support motor neuron survival and to favor nAChR clustering. Although the new protocol significantly supported myotube maturation and nAChR cluster differentiation, the amount of MYH1/α-actinin-positive cellular debris was increased in the d8 condition, likely representing remnants of well-differentiated myotubes that contracted prior to imaging. This is probably due to an accelerated maturation of cells that may contract powerfully and detach from the stiff substrate. This is in line with observations made by others (Osaki et al., 2018). Thus, although Matrigel provided a more physiological environment for myogenic differentiation, the plastic substrate was likely still too stiff for more long-term maturation, asking for a further adaption of the myogenic differentiation in a fully 3D and softer environment.

### 4.2 Motor neuron coculture reduces differences in nAChR cluster morphology between control and mutant monoculture myotubes

As *SOD1* D90A myotubes displayed a reduced myogenic marker expression compared to control, it was fitting to see that also aneural nAChR clusters of *SOD1* D90A myotubes differed morphologically from control ones. In fact, in monoculture, *SOD1* D90A cells displayed a reduced nAChR cluster solidity and an increased perimeter/area compared to control (Figures 4B–E). This finding fits to fragmented NMJs described in ALS mouse models (Clark et al., 2016; Dobrowolny et al., 2018b; Picchiarelli et al., 2019; Pereira et al., 2021; Mukhamedyarov et al., 2023; Tu et al., 2023). Next, we found that nAChR clusters of both, control and mutant myotubes, underwent a significant morphological remodeling upon coculture with motor neurons, i.e., a decrease in area and solidity and an increase in perimeter/area (Figures 4B–E). Yet, the postsynaptic remodeling upon motor neuron addition was less pronounced in mutant myotubes compared to controls. This rendered morphological features of nAChR clusters more similar between mutant and control cells, suggesting an involvement of neuronal factors in normalizing differences in the muscle-cell autonomous nAChR cluster formation. *In vivo*, the development of vertebrate NMJs is a step-wise process (Wu et al., 2010; Tintignac et al., 2015) that involves both aneural and neural components (Lin et al., 2008). First, with increasing differentiation, aneural myotubes show enhanced nAChR subunit expression and eventually, these prepattern in clusters. Subsequently, some clusters get contacted by motor neurons, often by several neurons at a time. Around birth, clusters usually appear as simple plaque-like structures that increasingly gain complexity by a concentration of nAChR in band-like arrangements that are interspersed by nAChR-free zones of sarcolemma (Slater, 1982). During this period of perinatal NMJ maturation, poly-innervation is eliminated by synaptic pruning and nAChR exhibit a subunit switch from embryonic α_2_βγδ to adult α_2_βεδ (Mishina et al., 1986; Witzemann et al., 1987). In mice, mature, healthy NMJs show nAChR in a typical pretzel-shaped pattern, in humans the pattern is sometimes less complex, but also showing specific gross morphological aspects (Flanagan-Steet et al., 2005; Kummer et al., 2006; Boehm et al., 2020). To achieve specialization of the postsynaptic apparatus of NMJs, at least three major processes are known to be at work. First, motor neurons induce a specialization of subsynaptic nuclei, whose transcriptional activity promotes expression of genes functioning in synaptic transmission (Simon et al., 1992; Ruegg, 2005; Tintignac et al., 2015; Saini et al., 2021). Second, cytoskeletal networks and associated organelles and proteins actively participate in postsynaptic organization, maturation and maintenance by transporting synaptic components to appropriate sites and by accumulating nAChR at the postsynaptic membrane (Sealock et al., 1989; Yorifuji and Hirokawa, 1989; Jasmin et al., 1990; Dai et al., 2000; Sanes and Lichtman, 2001; Kummer et al., 2006; Proszynski et al., 2009; Belhasan and Akaaboune, 2020; Lin et al., 2020; Osseni et al., 2020; Ghasemizadeh et al., 2021). Third, clustering and turnover of postsynaptic proteins, including nAChR, is regulated by neuronal signals and may be relayed intracellularly by second messenger signaling, protein phosphorylation and ubiquitination and regulation of postsynaptic protein trafficking and degradation (Martinez-Pena y Valenzuela and Akaaboune, 2021; Rudolf, 2023). Accordingly, nAChR cluster stability was addressed by analyzing nAChR internalization through a sequential αBGT live cell staining that allowed to visualize and quantify endocytic/lysosomal nAChR carriers. While *SOD1* D90A myotube monocultures featured an increased number of endocytic/lysosomal nAChR-positive puncta per muscle volume compared to controls, coculture with motor neurons significantly reduced these numbers for both cell lines, thus, partly reducing nAChR cluster stability differences observed between control and *SOD1* D90A (Figures 4F, G). It has remained elusive, if the increased number of endocytic/lysosomal nAChR-positive puncta was due to an overall enhanced nAChR turnover, as it is observed in denervated conditions in mice (Akaaboune et al., 1999; Tang and Goldman, 2006) or if a regular amount of endocytic nAChR was not sufficiently cleared as described upon a block of the autophagic/lysosomal degradation route (Carnio et al., 2014; Khan et al., 2014). Yet, the postsynaptic instability observed in ALS muscle cells is in accordance with observations made in ALS mouse models, displaying reduced endplate stability (Clark et al., 2016; Dobrowolny et al., 2018b; Badu-Mensah et al., 2022). Several pathways and organelles have been highlighted in NMJ integrity maintenance and ALS physiopathology and may support our observations. First, mitochondrial breakdown has been linked to oxidative stress and NMJ disruption (Chung and Suh, 2002; Dupuis et al., 2009; Dobrowolny et al., 2018b; Zhou et al., 2019; Belosludtseva et al., 2023). Another possible mode of action leading to NMJ instability in ALS could be a direct influence of the enzyme acetylcholinesterase (AChE). AChE contributes to neuromuscular transmission by its capacity to hydrolyze ACh, and therefore control synaptic ACh level, ensuring the functionality and the integrity of the synapse (Taylor and Radić, 1994; Dudel and Heckmann, 1999; Adler et al., 2004; Campanari et al., 2021). Downregulation of AChE in ALS motor endplates has been suggested to trigger its instability (Fernandez et al., 1986). Alternatively, Protein Kinase C (PKC) related pathways may support NMJ abnormalities in ALS. PKC has been demonstrated to be upregulated in ALS models and to trigger NMJ disintegration (Dobrowolny et al., 2018b; Camerino et al., 2019) and it is involved in nAChR clustering and NMJ formation (Lanuza et al., 2001; Lanuza et al., 2002; Martinez-Pena y Valenzuela et al., 2013).

### 4.3 Aberrant nAChR-cluster-dependent Ca^2+^ signaling in ALS muscle cells

Considering that muscle weakness and fatigability are major symptoms in ALS patients, Ca^2+^ transients as a key component underlying ECC were assessed in our model. ECC translates motor neuron impulses into muscle contraction (recently reviewed in Kaura and Hopkins, 2024). Although the difference was not statistically significant, our live myotube Ca^2+^-imaging reported a weaker nAChR-dependent Ca^2+^ response in mutant myotubes as compared to control myotubes, without any obvious correlation with nAChR cluster density (Figure 2; Supplementary Figure S1). This result is in accordance with an earlier study (Beqollari et al., 2016), that demonstrated reduced Ca^2+^ transient amplitudes in SOD1^G93A^ *ex vivo* myofibers using a voltage-clamp setup and which may be explained by a decreased affinity of ACh for its receptors (Palma et al., 2011; Palma et al., 2016) or by other downstream alterations. Indeed, modification of ECC events were also observed in *SOD1*-related ALS mouse models. As such, EDL muscles of SOD1^G93A^ mice displayed a downregulation of Nav1.4 voltage-gated sodium channel transcripts associated to a decreased amplitude of the action potential and to impaired sarcolemmal excitability (Camerino et al., 2019). Additionally, an uncoupling of triad junctions with transverse tubules was suggested to amplify the excitation-contraction coupling impairment in SOD1^G93A^ mice (Dobrowolny et al., 2008). Within the t-tubules of SOD1^G93A^ mice, downregulation of voltage-gated L-type Ca^2+^ channels (Beqollari et al., 2016) and of Ryanodine receptor 1 (Camerino et al., 2019) were reported and associated with a decreased Ca^2+^ release from the sarcoplasmic reticulum. This contrasts with other reports demonstrating an increase in depolarization-dependent Ca^2+^ transients in FDB fibers from SOD1^G93A^ mice (Zhou et al., 2010; Yi et al., 2011). Some ALS-related alterations appeared to be fiber type-specific, such as differential protein expression (i.e., dihydropyridine receptors) (Delbono and Meissner, 1996; Payne and Delbono, 2004) and adaptation of muscle metabolism (Smittkamp et al., 2014). In view of the ratio variability between slow and fast fiber types amongst muscles and of a fast-to-slow phenotype transition described in SOD1^G93A^ mice (Dobrowolny et al., 2018a; Camerino et al., 2019), contradictory results may arise and accurate comparison between studies remains delicate.

Next, to decipher which proportion of ACh-induced calcium transients was nAChR dependent, we pretreated myotubes with αBGT prior to ACh stimulation. αBGT induces a conformational arrest of the nAChR α1-subunit binding sites via a non-competitive and dominant effect (daCosta et al., 2015). Interestingly, αBGT pre-treatment reduced the ACh-induced Ca^2+^ transients more strongly in control than in mutant myotubes (45.2% vs. 18.6%, Figures 2A,B’; Supplementary Figure S1), such that the residual transients were now very similar between both cell types. This suggests two things: first, that, comparing control and mutant cells, the Ca^2+^ mobilization relied to a different extent on nAChR and, second, that in both cell types there might be another ACh-dependent but nAChR-independent Ca^2+^ component. The first aspect was already discussed above. As for the second Ca^2+^ component, this might be explained by activation of muscarinic acetylcholine receptors (mAChR). mAChR are classified into five different subtypes; amongst these M1, M3 and M5 are expressed in skeletal muscle fibers and preferentially activate Gq/G11-type G-proteins (daCosta et al., 2015). Muscarinic AChR downstream effectors have been strongly implicated in muscle growth and atrophy (Wright et al., 2009), and their dependent signaling pathways might be important not only in the early developmental stages of the muscle cells, but also after denervation of adult muscle fibers (Furlan and Godinho, 2005). mAChR synthesis was suggested to be characteristic of aneural developing muscle fibers (Furlan and Godinho, 2005). Further experiments utilizing antagonists of mAChR would be necessary to verify this hypothesis.

## 5 Conclusion

The described human NMJ model is the first to show complex nAChR clusters *in vitro* with the option of further maturation in a coculture setup with iMN. This model showed an altered nAChR-dependent Ca^2+^ response in *SOD1* D90A mutant myotubes, supporting the idea of an impaired excitation-contraction coupling in ALS-muscle. Furthermore, the study identified reduced sarcomeric organization and limited motor neuron-induced postsynaptic plasticity in mutant myotubes. Trophic support provided by iMN induced a significant nAChR cluster remodeling, and reduced nAChR cluster differences observed in monocultures between the two lines.

## Data Availability

The raw data supporting the conclusions of this article will be made available by the authors, without undue reservation.

## Abbreviations

αBGT, α-bungarotoxin; ACh, acetylcholine; ALS, amyotrophic lateral sclerosis; ECC, excitation-contraction coupling; hiPSC, human induced pluripotent stem cell; iMN, hiPSC-derived motor neurons; MND, myonuclear domain; MYH1, Myosin Heavy Chain 1; MyoG, Myogenin; nAChR, nicotinic acetylcholine receptors; NMJ, neuromuscular junction; RT, room temperature; SMCGM, skeletal muscle cell growth medium.
