# Supplementary material for: Aberrant evoked calcium signaling and nAChR cluster morphology in a SOD1 D90A hiPSC-derived neuromuscular model
Source: Front Cell Dev Biol. 2024 Jun 20;12:1429759. doi: 10.3389/fcell.2024.1429759 (PMC11222430; doi:10.3389/fcell.2024.1429759)
Supplement: Supplementary file 4 [file Table1.DOCX]

Supplementary Material to Manuscript:

Aberrant evoked calcium signaling and nAChR cluster morphology in a SOD1 D90A hiPSC-derived neuromuscular model; Couturier et al.

# Supplementary Figures

## Supplementary Figure 1:


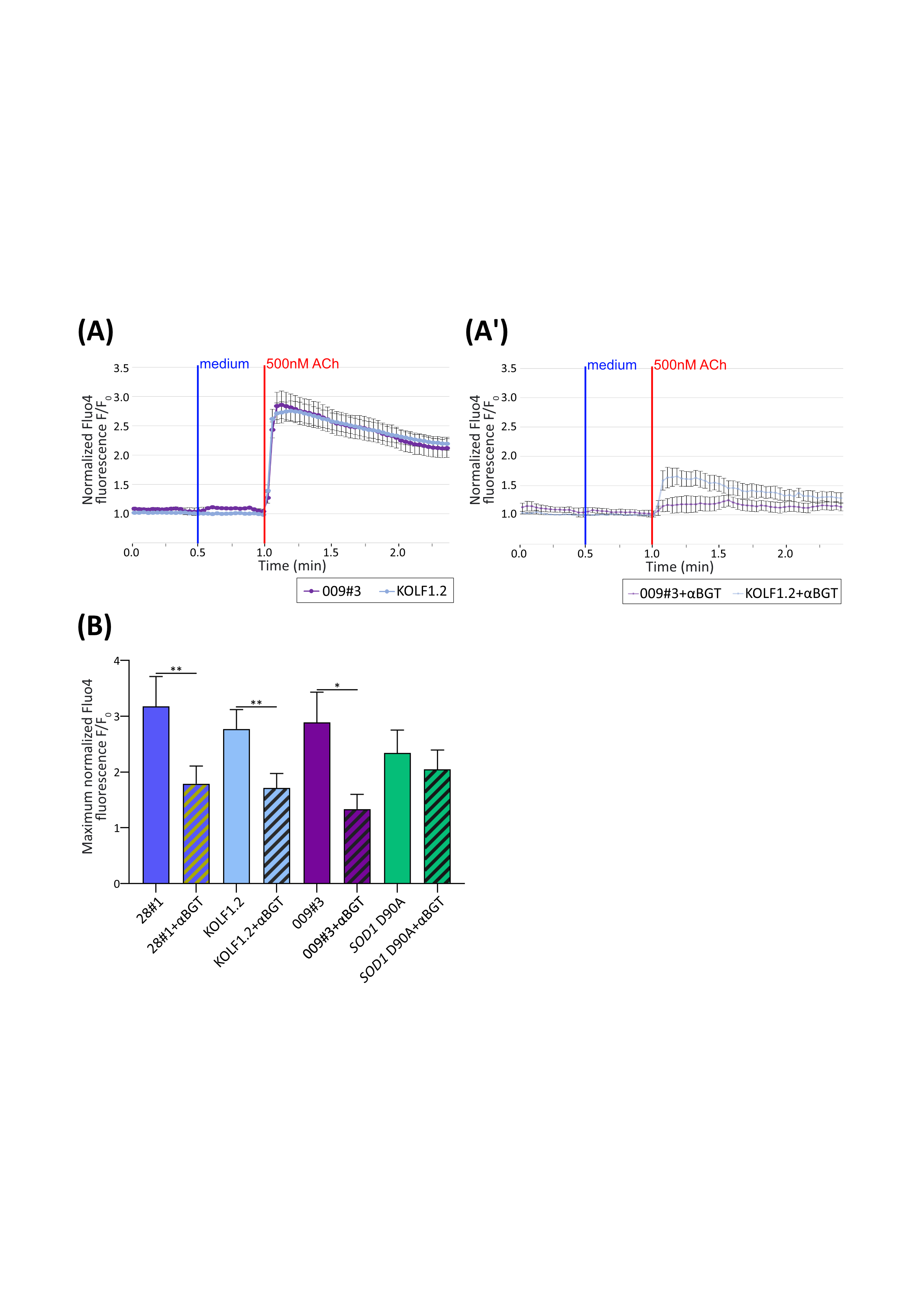


**Supplementary Figure 1**: hiPSC-derived myotube cultures differentiated for 50+4 days were subjected to Fluo4-mediated Ca^2+^ imaging in the presence of 500nM ACh ± αBGT ([3µg/mL]). Myotubes were stimulated with ACh either directly (A) or after pre-treatment with αBGT (A’). (A) ΔF/F_0_ Fluo4 kinetics for KOLF1.2 and 009#3 myotubes upon ACh stimulation, without (A) and with (A’) αBGT pre-treatment. Fluo4 fluorescence was normalized to corresponding baseline values. Curves depict mean ± SE of at least three biological replicates. (B) Maximum normalized Fluo4 fluorescence at peak upon ACh. *p < 0.05; **p < 0.01.

## Supplementary Figure 2


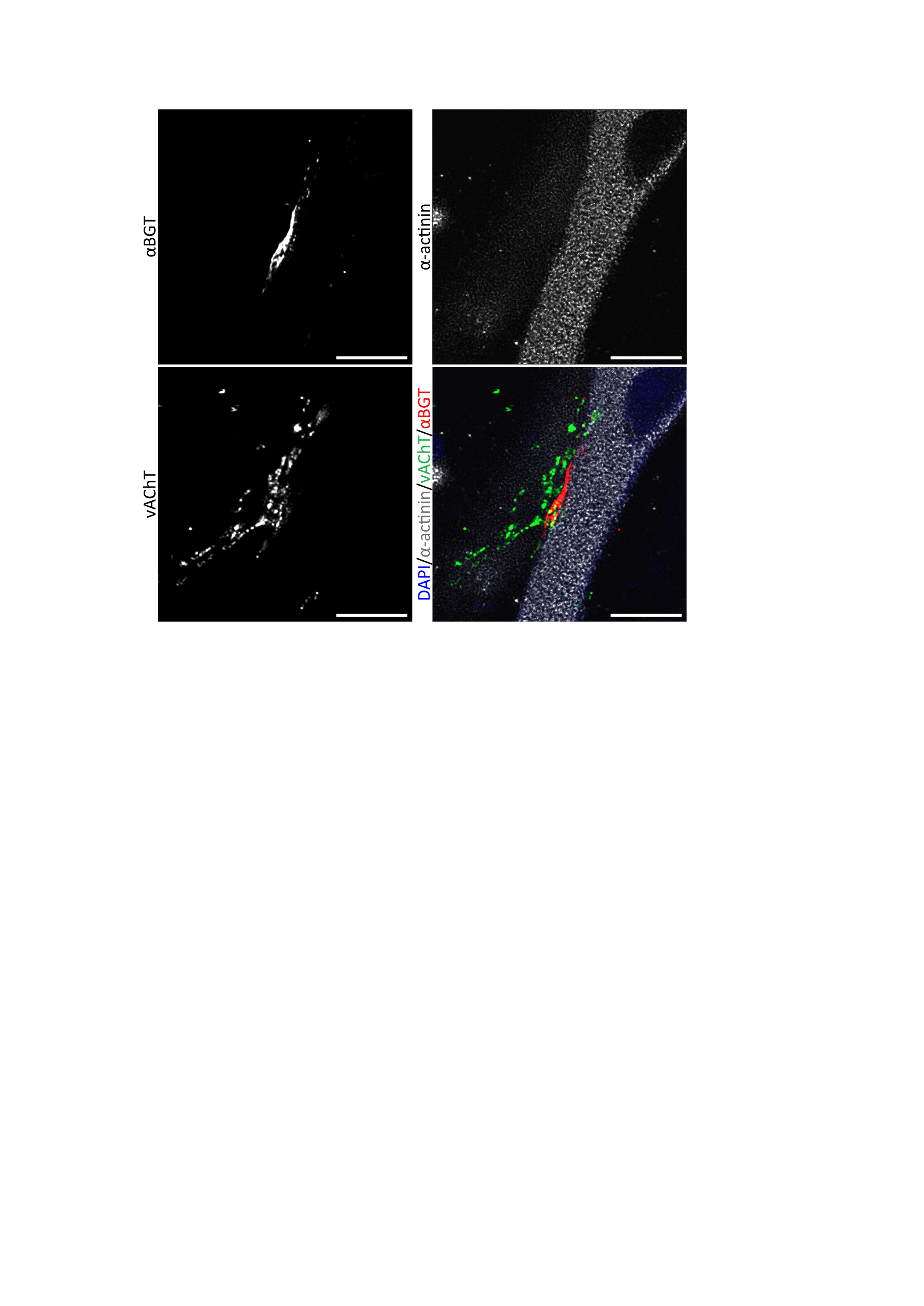


**Supplementary Figure 2:** Myoblasts were expanded for 6 days (d-6 to d0), further differentiated for 8 days (d0 to d8) and cocultured with control iMN. Fluorescence signals show nuclei (blue), αBGT-stained nAChR clusters (red), α-actinin (grey) and vesicular acetylcholine transport (vAChT, green). Maximum projection of 1.5µm. Scale bars, 10µm.

# Supplementary Videos

## Supplementary Figures

**Supplementary Videos 1 - 4:** Time-lapse videos d4 differentiated myotubes in control (supplementary videos 1 and 3) and *SOD1* D90A (supplementary videos 2 and 4) conditions, with (supplementary videos 3 and 4) and without (supplementary videos 1 and 2) αBGT pre-treatment. N2-based medium and 500nM ACh were added to the cultures as indicated on the videos. Time interval between consecutive frames, 2s. Scale bars, 100 µm.

**Supplementary Video 5:** 3D-rendering of d8-control myotubes. Striated pattern of myotubes is highlighted by sarcomeric α-actinin (grey) and nAChR clusters with an αBGT staining (magma). 3D-rendering was generated from a z-stack of 16 µm thickness. Images were processed as described in materials and methods section. Scale bar is adjusted according to zoom shown in the animation.

**Supplementary Video 6:** Video highlighting pre- and post-synaptic elements in d8-control myotubes cocultured with iMN. Pre-synapse represented by neurites emanating from iMN were stained for vAChT (green), post-synapse corresponding to nAChR clusters are visualized by a αBGT staining (red), and myotubes are stained for α-actinin (grey). Video covers a thickness of 18 µm of a z-stack image. Images were processed as described in materials and methods section. Scale bar is adjusted according to zoom shown in the video.
